# A Study on Hollow Mesoporous Silica Nanoparticles with Long-Term Cycling

**DOI:** 10.3390/ma18245618

**Published:** 2025-12-15

**Authors:** Min su Kim, Jung hun Lee, In-Bo Shim

**Affiliations:** 1 Department of Nano & Electronic Physics, Kookmin University, Seoul 02707, Republic of Korea; david714150@gmail.com; 2 Department of Physics, Kookmin University, Seoul 02707, Republic of Korea; l00l4032@kookmin.ac.kr

**Keywords:** hollow mesoporous silica, anode materials, lithium-ion batteries, cycling stability, SEI formation

## Abstract

As electronic technologies continue to advance, the demand for high-performance and safe batteries has steadily increased. However, silicon-based anode materials experience severe volume expansion and poor structural stability during cycling, which limits their practical application. In this study, we synthesized hollow mesoporous silica to develop an anode material with long-term cycling stability. Electrochemical analysis revealed that the material exhibited low-capacity decay, decreasing from 125 mA·h·g^−1^ to 120 mA·h·g^−1^ at a C-rate of 20 C, and retained a 49 mA·h·g^−1^ after 500 charge–discharge cycles at a C-rate of 10 C. Furthermore, electrochemical impedance spectroscopy and Scanning Electron Microscopy analysis confirmed that the hollow mesoporous silica structure is long-term cycling stability in the anode.

## 1. Introduction

Energy storage devices are important components for operating portable electronic equipment such as smartphones, laptops, and smartwatches. As electronic device technologies continue to advance, the demand for high-performance batteries has steadily increased [1,2]. Accordingly, research of and demand for various energy storage systems—including solid-state batteries, solar cells, and lithium-ion batteries—have also grown, highlighting the need for technological innovation and advancement. However, significant safety and stability concerns remain inherent to these devices. In particular, lithium-ion batteries are prone to fires caused by short circuits and overheating due to dendrite formation, which remain among the most critical challenges to overcome [3]. To address these safety issues in high-performance batteries, innovation in battery materials has become an essential research priority.

Meanwhile, a major issue encountered during the charge–discharge processes of high-capacity materials commonly used in secondary batteries, such as Si and Ge, is volume expansion. In the case of Si, the volume expansion can reach up to 300% of the original size, which hinders the formation of a stable solid electrolyte interphase (SEI) layer [4,5]. To address this issue, recent studies have focused on improving the electrochemical performance by synthesizing carbon coatings to ensure mechanical stability against volume expansion, forming core–shell structures, or compositing with carbon nanotubes (CNTs) and graphene [6]. However, these approaches have limitations in fundamentally enhancing the intrinsic stability of high-capacity materials.

A comparison with commonly used anode materials, such as graphite and silicon, can provide a clearer understanding of the advantages and limitations of SiO_2_. Graphite, a commercialized anode material, has advantage due to its long life cycle, safety, and low cost; however, its primary limitation is a low theoretical capacity of 372 mA·h·g^−1^. Si anodes have the advantage of a high theoretical capacity 4200 mA·h·g^−1^, low discharge potential, and abundant resources. SiO_2_ anodes exhibit smaller volume expansion and better chemical stability than Si. Nevertheless, they have disadvantages such as a lower theoretical capacity of 1965 mA·h·g^−1^ compared to Si and poor electrical conductivity [7].

In the case of SiO_2_, various synthesis methods enable the formation of multilayered structures while retaining the intrinsic advantages of the material. Notably, the Stöber method allows facile synthesis of silica with porous or hollow structures [4,8]. Moreover, SiO_2_ is chemically stable and resistant to side reactions during charge–discharge processes, making it a promising anode active material in terms of stability.

Recent studies have employed compositing and various structural designs to overcome the limitations of the theoretical capacity of SiO_2_ [8,9]. Among these strategies, mesoporous structures offer advantages in accommodating volume expansion and providing a favorable reaction surface area during charge–discharge cycles. Furthermore, investigations focusing on mesoporous silica structures have demonstrated improved electrochemical properties due to enhanced charge transfer capabilities [10].

In particular, Wang et al. [11] demonstrated that mesoporous structures can significantly enhance electrochemical performance, achieving a capacity of 1060 mA·h·g^−1^ after 90 cycles at a current density of 100 mA·g^−1^. Similarly, Peng et al. [12] reported that mesoporous SiOx delivers a capacity of 1434 mA·h·g^−1^ with 67.5% capacity retention after 500 cycles at 500 mA·g^−1^, outperforming conventional SiO_2_. Recent studies have focused on nanoscale silica nanostructures as anode materials, which exhibit superior properties compared to conventional SiO_2_ nanoparticles. For example, hollow structures synthesized without templates show excellent long-term cycling stability [13]. Additionally, densification of graphite-silica composites via ball milling has been reported to achieve an initial Coulombic efficiency of 73%, a specific capacity of 491.9 mA·h·g^−1^ at 5 A·g^−1^, and a stable capacity of 600.3 mA·h·g^−1^ after 500 cycles at 0.5 A·g^−1^ [14].

In addition, recent studies have reported that higher stability and efficiency can be achieved by modifying the active material structure, even without a composite. Nan et al. [15] synthesized hollow porous SiO_2_ nanocubes, which maintained a high reversible capacity of 919 mA·h·g^−1^ after 30 charge–discharge cycles and maintained 377 mA·h·g^−1^ after 25 cycles at a high current density of 500 mA·g^−1^. Miaolun et al. [16] synthesized a hollow SiO_2_/Carbon composite, which maintains a high reversible capacity of 669.8 mAh·g^−1^, 98.6% capacity retention, and 99.5% Coulombic efficiency after 100 charge–discharge cycles.

This study is focused on the structural characteristics of HMSNs, which have been previously investigated as drug delivery carriers, to propose the potential application of their hollow mesoporous architecture as an active material for anodes [17]. In addition, considering the structural benefits of the hollow and mesoporous frame works such as enhanced stability during charge–discharge cycling, favorable lithium-ion diffusion, and high electronic conductivity electrochemical properties and EIS analyses for long-term cycling performance are presented.

## 2. Materials and Methods

### 2.1. Materials

D-(+)-Glucose (Sigma Aldrich, St. Louis, MO, USA, G5767, ACS reagent) was used as a precursor for the synthesis of carbon nanoparticles. For the silica synthesis, (3-Aminopropyl)triethoxysilane (APTES) (Sigma Aldrich, 440140, 99%) and Tetraethoxysilane (TEOS) (Sigma Aldrich, 131903, 98%) were used. Ammonium hydroxide solution (Samchun Chemical, Seoul, Republic of Korea, 28~30%) was used as a catalyst. Cetrimonium bromide (CTAB) (Sigma Aldrich, H5882, 98%) was used as a synthesis additive. Sodium hydroxide (NaOH) (Sigma Aldrich, 415413, 50% solution in water) was used for pH control. For electrode preparation, Poly(acrylic acid) (PAA) (Sigma Aldrich, 523925, 35 wt% in H_2_O), Super P (Thermo Fisher Scientific, Waltham, MA, USA, H30253.14, 99+%), lithium chip, a Celgard separator, and an electrolyte (1 M LiPF_6_ in EC/DEC with 10% FEC additive) were used.

### 2.2. Synthesis of Carbon Nanoparticles (CNPs)

To synthesize the carbon nanoparticles (CNPs), 12 g of glucose was dissolved in 120 mL of deionized water. The resulting solution was then placed into an autoclave and subjected to hydrothermal treatment at 180 °C for 2 h, with a heating rate of 2 °C/min.

### 2.3. Synthesis of Hollow Mesoporous Silica Nanoparticles

For the synthesis of HMSNs, 0.05 g of the as-prepared CNPs were dispersed in 20 mL of ethanol, followed by the addition of 85 μL of APTES. After stirring this mixture, 85 μL of TEOS and 250 μL of ammonium hydroxide (NH_4_OH) were added, and the solution was stirred for 12 h [18,19]. The resulting product was collected by centrifugation, the supernatant was discarded, and the precipitate was redispersed in 6 mL of deionized water via sonication. To this dispersion, 12 mL of additional deionized water, 170 μL of TEOS, and 0.1 mol of CTAB were added and stirred for 10 min to form the mesoporous shell. After adjusting the pH to 10.5–11.5 with NaOH, the reaction was continued at 55 °C for 2 h. The synthesized powder was then washed three times each with deionized water and ethanol and dried in vacuum oven at 60 °C for 24 h. Finally, the HMSNs were obtained by calcining the powder in air at 550 °C for 5 h at a heating rate of 3 °C/min.

### 2.4. Electrode Preparation

To prepare the anode for the half-cell, a slurry was formed by mixing HMSNs, Super P, and PAA in a weight ratio of 7:2:1 with deionized water. The slurry was then cast onto a copper foil using a doctor blade and dried at 100 °C for 18 h. Galvanostatic charge–discharge profiles, rate capability electrode mass loading 2.12 mg/cm^2^, cycling performance electrode mass loading 2.20 mg/cm^2^, electrochemical impedance spectroscopy (EIS) data electrode mass loading 2.12 mg/cm^2^.

### 2.5. Characterization

The crystal structure of the synthesized HMSNs was examined by powder X-ray diffraction (XRD; RIGAKU Corporation, Akishima, Tokyo, Japan, Ultima IV) in the 2θ range of 10–60°, using Cu Kα radiation with a characteristic wavelength of 1.5418 Å at a scan rate of 4°/min. The microstructures and compositions of the CNPs and HMSNs were examined using Scanning Electron Microscopy (SEM; HITACHI High-Tech Corporation, Tokyo, Japan, SU8700) and high-resolution transmission electron microscopy (HR-TEM; JEOL Ltd., Akishima, Tokyo, Japan, JEM-3010). Electrochemical performance was evaluated using CR2032 coin-type half-cells. Galvanostatic charge–discharge (GCD) profiles and C-rate capabilities were measured using a battery cycler (Won-A Tech Co., Ltd., Siheung-si, Gyeongggi-do, Republic of Korea, WBCS3000) within a voltage window of 0.05–2.85 V (vs. Li/Li^+^), where 1C corresponds to a current density of 100 mA·g^−1^. Cell impedance was analyzed by electrochemical impedance spectroscopy (EIS; IVIUM Technologies B.V., Eindhoven, The Netherlands, Ivium-n-Stat) over a frequency range of 200 kHz to 0.01 Hz, with an applied voltage of 0.01 V and a current of 10 mA.

## 3. Results

**Figure 1:**
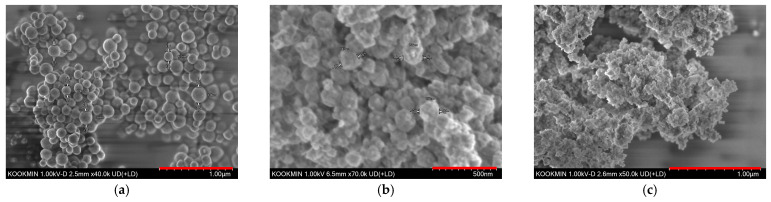
(**a**) SEM image of CNPs synthesized via the hydrothermal method. (**b**) SEM image of HMSNs after 550 °C calcination. (**c**) SEM image of HMSNs after 500 °C.

The crystal structure of the HMSNs was analyzed by XRD. In Figure S1, the resulting pattern displayed a broad halo peak around 22.4° which is characteristic of amorphous silica [20]. Figure 1 shows the SEM images of the CNPs synthesized via a hydrothermal method and the HMSNs synthesized via the Stöber method. Figure 1a reveals that the CNP template consists of spherical particles with diameters of approximately 180–200 nm. Figure 1b,c display the SEM images of the HMSNs after calcination at 550 °C and 500 °C, respectively, showing a distinct wrinkled surface morphology. This rough surface suggests the formation of a porous surface, a finding consistent with the work of Xiong et al. [21]. The resulting HMSNs were relatively uniform in size, with diameters of about 190–210 nm. Table 1 presents the EDS data for the samples calcined at 550 °C and 500 °C. A decrease in carbon content of 5.19 wt% was confirmed for the sample calcined at 550 °C.

**Figure 2:**
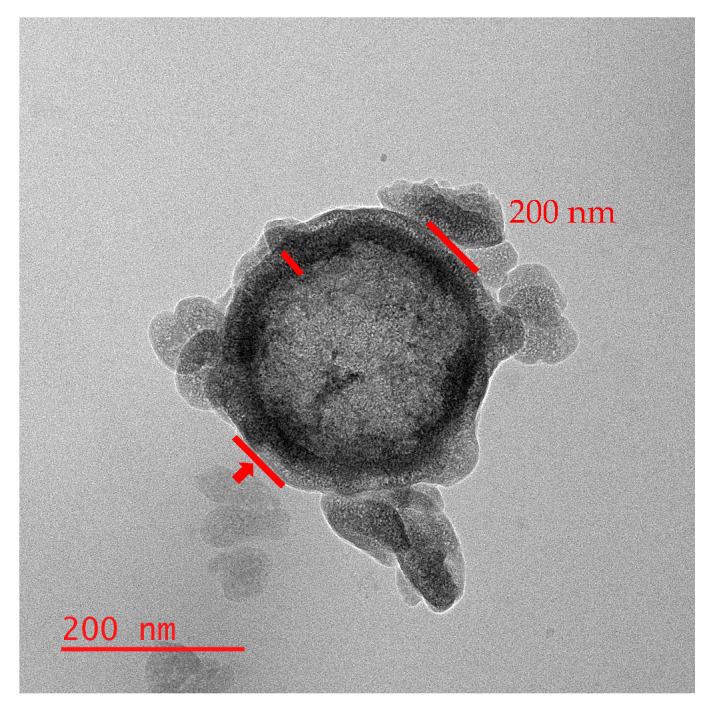
TEM images of the HMSNs after calcination.

Figure 2 presents the TEM images of the HMSNs synthesized via the Stöber method, which confirm the structure and size of the particles. The images clearly reveal that the synthesized silica possesses a hollow structure [22]. The TEM analysis also reconfirmed that the particles have a diameter of approximately 190–210 nm. As indicated in the images, the silica shell thickness was measured to be around 10–15 nm. Furthermore, the varying contrast in Figure 2 suggests that the particles have a porous surface [23].

**Figure 3:**
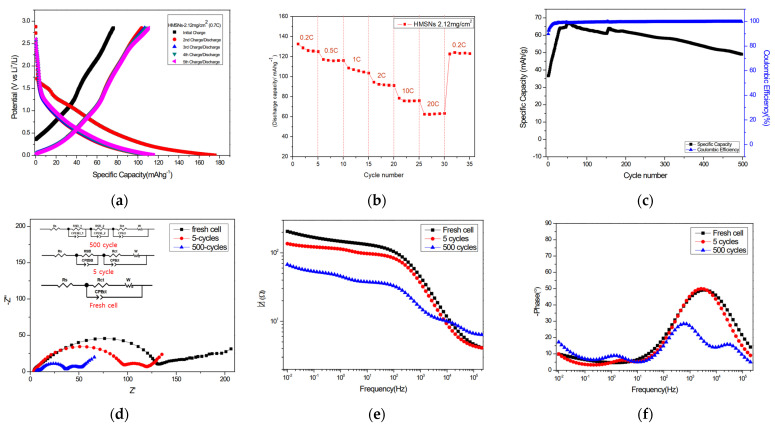
Lithium storage measurement of SiO_2_ in a half cell. (**a**) Galvanostatic charge–discharge profiles, (**b**) rate capability, (**c**) cycling performance, (**d**) Nyquist plot, (**e**) bode-mode plots, (**f**) Bode-phase plots.

Figure 3 details the electrochemical performance of the HMSN anode in a half-cell configuration, tested within a voltage window of 0.005–2.85 V (vs. Li/Li^+^) The electrochemical performance was evaluated at a current density corresponding to a C-rate of 100 mA·g^−1^. Figure 3a presents the GCD profiles for the initial five cycles. Figure 3b illustrates the rate capability of an electrode with a mass loading of 2.12 mg/cm^2^ at various C-rates from 0.2 C to 20 C. The long-term cycling stability of an electrode with a 0.57 mg/cm^2^ mass loading is shown in Figure 3c, which was tested at a high rate of 12 C for 1000 cycles.

As shown in the GCD profile in Figure 3a, tested at 0.7 C, the anode delivered a high initial charge capacity of 175 mA·h·g^−1^. This is followed by a stable charge capacity of 110 mA·h·g^−1^ from the second to the fifth cycle. The initial discharge capacity was 67 mA·h·g^−1^. The large irreversible capacity loss after the first cycle is primarily attributed to the formation of a stable SEI layer on the electrode surface [24,25,26]. As shown in Figure 3b, electrode delivered stable capacities of approximately 125–132 mAh·g^−1^ at 0.2 C 1–5 cycles. The electrode capacity remained 60 mAh·g^−1^ at a high rate of 20 C. When the C-rate was reverted to 0.2 C, the capacity was approximately 120 mA·h·g^−1^. It was confirmed that it was similar to the initial 0.2 C capacity.

As shown in Figure 3c, the electrode approaches a capacity of approximately 60 mA·h·g^−1^ in 55 cycles at a rate of 10 C. It was confirmed that it was similar to the initial capacity. Slight increase in capacity of about 30 mA·h·g^−1^ from the initial value was observed as cycling progressed. This phenomenon has been reported in the literature. Favors et al. [24] propose a mechanism wherein SiO_2_ is partially reduced by lithium, leading to the growth of a Si phase. The irreversible formation of Li_4_SiO_4_ at the interface therefore creates triply bonded Si (III). The capacity gain associated with this process is greater than the capacity loss from the consumption of SiO [25,27]. The lithiation process of SiO_2_, the formation of Li_4_SiO_4_ and the growth of Si, are described in Equations (1a)–(1c)

(1a) $$SiO_{2}+4Li^{+}+4e^{-}\to Si+2Li_{2}O$$

(1b) $$4Li^{+}+2SiO_{2}+4e^{-}\to Li_{4}SiO_{4}+Si$$

(1c) $$xLi^{+}+Si+xe^{-}↔Li_{x}Si$$

where the capacity slightly decreased during the 500 cycles. The capacity was about 49 mA·h·g^−1^ at 500 cycles. Nonetheless, the decrease was quite stable in harsh and long-cycled electro-chemical measurements. Also, the Coulombic efficiency reached 99.85% at the 500th cycle, demonstrating the excellent stability of the HMSNs during long-term charge–discharge processes. Figure 3d shows the Nyquist spectra for the anode half-cell at its pristine state (0 cycles), and after 5 and 500 cycles. Notably, the Nyquist plot after 500 cycles reveals the formation of three distinct semicircles, in contrast to the two observed after 5 cycles. This phenomenon is attributed to the formation of a bi-layer SEI on the surface of the porous material [26]. The EIS data were fitted using equivalent circuits. For the fresh cell, where no SEI is formed, the circuit consists only of the solution resistance (R_s_), charge transfer resistance (R_ct_), Warburg impedance (W), and a constant phase element (CPE_ct_). After 5 cycles, an additional parallel circuit of R_SEI_ and CPE_SEI_ was introduced to account for the initial formation of the SEI layer. To model the multi-layered structure after 500 cycles, a third semicircle was added, represented by a second R_SEI_-CPE_SEI_ parallel circuit [28]. The fitted resistance values at each cycle, as summarized in Table 2, were as follows: at 0 cycles, R_s_ = 3.512 Ω and R_ct_ = 122.6 Ω; at 5 cycles, R_s_ = 3.792 Ω, R_SEI_ = 95.94 Ω, and R_ct_ = 11.2 Ω; and at 500 cycles, R_s_ = 6.254 Ω, R_SEI_1_ = 3.496 Ω, R_SEI_2_ = 27.97 Ω, and R_ct_ = 9.461 Ω. The R_s_ value shows little change between 0 and 5 cycles but increases by 2.462 Ω after 500 cycles, which is likely due to electrolyte degradation over extended cycling. Conversely, the R_ct_ value decreased as cycling progressed, indicating that ion transfer and electrochemical reactions at the interface became more facile [29]. In Figure 3e, the logarithmic impedance in the high-frequency region increases in the order of the fresh cell, 5-cycle cell, and 500-cycle cell. This region mainly reflects the series resistance (R_s_), indicating that the gradual rise in R_s_ with cycling is associated with electrolyte changes occurring during repeated lithiation and delithiation, thereby revealing progressive cell aging. In contrast, the low-frequency region shows a decreasing trend in the logarithmic impedance from the fresh cell to the 5-cycle and 500-cycle cells. This behavior signifies a reduction in charge-transfer resistance (R_ct_) as cycling progresses, suggesting that ion-transport resistance at the electrode interface becomes more stabilized during repeated charge–discharge processes. The presence of the first semicircle for the 500-cycle cell in the high-frequency range of the Nyquist plot can be corroborated by the initial slope observed in the 500-cycle spectrum of Figure 3e. Additionally, Figure 3f shows an inflection point near 30,000 Hz, which further supports this feature. For the 500-cycle cell, three downward inflection points appear at approximately 8.8 kHz, 11.9 Hz, and 0.19 Hz, corresponding to the characteristic frequencies of R_SEI1_, R_SEI2_, and R_ct_, respectively. Similarly, in the spectra of the 5cycle and fresh cells, the characteristic frequencies associated with R_SEI1_ and R_ct_ can be identified at around 8.77 Hz and 0.20 Hz for the 5-cycle cell.

**Figure 4:**
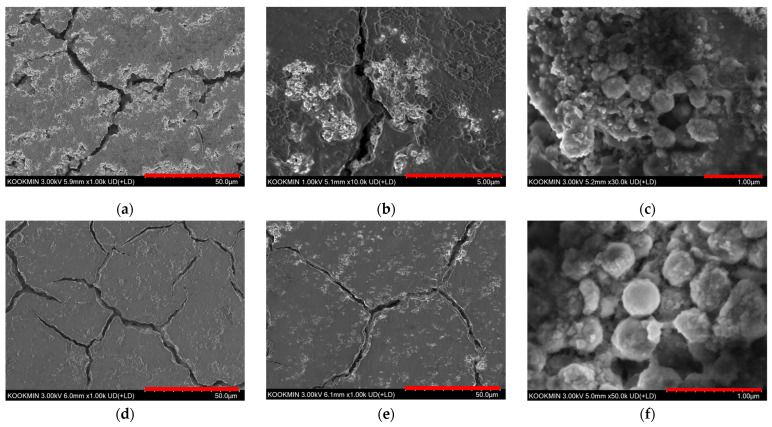
SEM images of the electrode after cycles. (**a**–**c**) After 5 cycles at 0.1 C, and (**d**–**f**) after 500 cycles at 10 C.

Figure 4a–c display the SEM images of an electrode after 5 cycles at 0.1 C. As shown in Figure 4a,b, a comparison with the pristine HMSN in Figure 1b,c reveals the formation of a thin SEI layer during the initial cycling process. Importantly, the spherical morphology of the HMSNs was well-maintained as seen in Figure 4c. Figure 4d–f show the electrode surface after 500 cycles at a high rate of 10 C. Compared to the 5-cycle electrode, a more uniform and stable SEI layer is evident in Figure 4d,e. Although minor structural alterations to some HMSNs are visible, the majority of particles retained their original shape as seen in Figure 4f. Therefore, the collective EIS and SEM analyses confirm that HMSNs possess outstanding long-term charge–discharge stability.

In this study, HMSNs were synthesized using the Stöber method to be developed as an anode material with long-term cycling stability. Electrochemical analysis of the HMSNs revealed negligible capacity difference at 0.2 C after cycling at 20 C and stably maintained capacity without degradation after 1000 cycles. Furthermore, EIS analysis showed a slight decrease in the R_ct_ value, which indicates that ion transfer and electrochemical reactions remained stable and SEM analysis confirmed the preservation of the particle morphology and the formation of a stable SEI layer. Consequently, these results verify that HMSNs are anode material with exceptional long-term cycling stability.

## 4. Discussion

When compared with the reference studies summarized in Table 3, the HMSNs exhibit a clear limitation in terms of specific capacity. Nevertheless, after 500 charge–discharge cycles under a high current density of 1 A·g^−1^, the material maintains a Coulombic efficiency of 99.85%, which exceeds both the current densities and Coulombic efficiencies reported in the cited literature. These results suggest that the intrinsic structural features of the HMSNs contribute substantially to their long-term cycling stability. This observation further implies that other silica-based carriers developed for drug-delivery applications, if they possess comparable structural robustness, may also be repurposed as anode materials for lithium-ion batteries.

Although the present material system still shows limitations in achievable capacity, it appears to have the potential for further improvement through more deliberate tuning of cavity dimensions, shell thickness, and mesopore size, as well as through composite strategies incorporating carbonaceous components. From this perspective, HMSN-based architectures represent a promising platform that could be further advanced through future structural and compositional design efforts aimed at enhancing overall electrochemical performance.

## 5. Conclusions

In this study, HMSNs was synthesized using the Stöber method to be developed as an anode material with long-term cycling stability. The electrochemical analysis of the HMSNs revealed stable charge–discharge performance at high current conditions, as indicated by the low-capacity decay, decreasing from 125 mA·h·g^−1^ to 120 mA·h·g^−1^ at 0.2 C after cycling at 20 C. The electrode also exhibited stable performance at high rates, reaching a capacity of approximately 60 mA·h·g^−1^ after 55 cycles at 10 C, demonstrating excellent long-term cycling stability. EIS analysis and post-cycling SEM confirmed the formation of a stable SEI and the preservation of the particle morphology. This indicates that the material undergoes stable electrochemical cycling without structural deformation. Therefore, it was confirmed that HMSNs are anode materials with exceptional long-term cycling stability.

## Figures and Tables

**Table 1:** EDS data of HMSNs calcined at 500 °C and 550 °C.

Element	500 °C (wt%/Atomic%)	550 °C (wt%/Atomic%)
C	10.01/15.90	4.82/8.07
O	44.82/53.43	44.20/55.49
Si	45.17/30.67	50.97/36.45

**Table 2:** Fitted resistance parameters from EIS data at different cycle numbers.

	R_s_ (Ω)	R_ct_ (Ω)	R_SEI_ (Ω)	CPE_SEI_ (F)	CPE_ct_ (F)
Fresh cell	3.512	122.6	-	-	0.7227
5 cycle	3.792	11.2	95.94	0.77	1.15
500 cycle	6.264	9.461	3.496/27.97	0.999/0.8372	1.013

**Table 3:** Variation in electrochemical performance of SiO_2_ materials.

Anode	Current Density (mA·g^−1^)	Initial CE (%)	Reversible Capacity (mA·h·g^−1^)	After nth Cycle (Cycle)	Reference
Mesoporous SiO_2_ nanoparticles	100	17.4	1060	90	[11]
m-SiOx/C	300	66.8	613	200	[12]
Hollow porous SiO_2_ nanocubes	100	47	919	30	[15]
Hollow SiO_2_/carbon	100	48.2	649.6	160	[16]
HMSNs	1200	43.4	80	1000	

## Data Availability

The original contributions presented in this study are included in the article/Supplementary Materials. Further inquiries can be directed to the corresponding author.

## Supplementary Materials

The following supporting information can be downloaded at https://www.mdpi.com/article/10.3390/ma18245618/s1, Figure S1. X-ray diffraction (XRD) data of HMSNs.

## Abbreviations

EIS	Electrochemical Impedance Spectroscopy
SEM	Scanning Electron Microscopy
EC	ethylene carbonate
LIB	Lithium-Ion Battery
HMSNs	Hollow Mesoporous Silica Nanoparticles
APTES	(3-Aminopropyl)triethoxysilane
CTAB	Cetrimonium bromide
NaOH	Sodium hydroxide
PAA	Poly(acrylic acid)
CNPs	carbon nanoparticles
GCD	Galvanostatic charge–discharge
SEI	Solid Electrolyte Interphase
TEM	high-resolution transmission electron microscopy
CNTs	carbon nanotubes
