# Supplementary figures and images for: A Study on Hollow Mesoporous Silica Nanoparticles with Long-Term Cycling
Source: Materials (Basel). 2025 Dec 15;18(24):5618. doi: 10.3390/ma18245618 (PMC12734838; doi:10.3390/ma18245618)

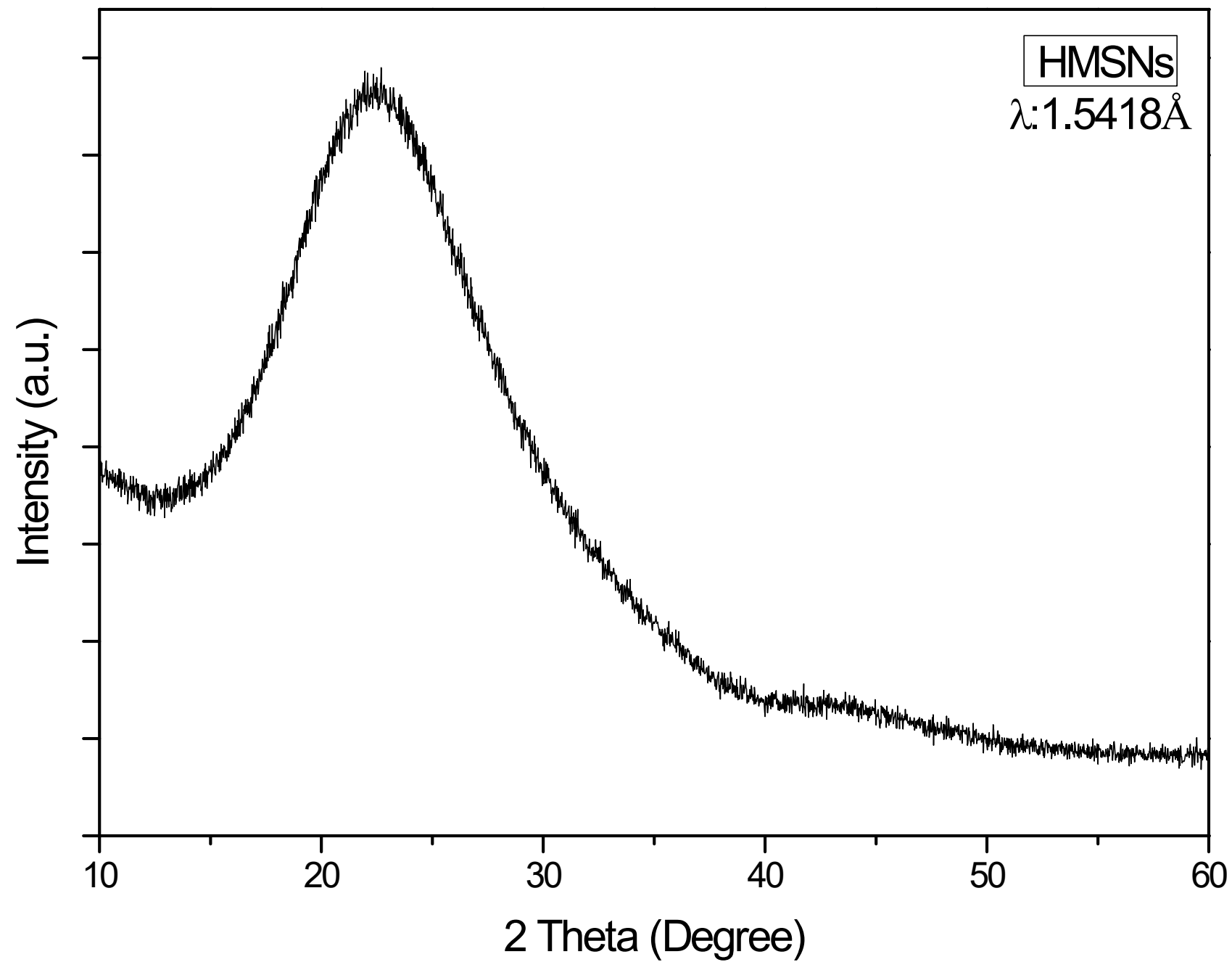

Supplement: Supplementary file 1 [file materials-18-05618-s001.zip › materials-3976005-supplementary.pdf]
